# Duck Hepatitis A Virus Type 1 Induces eIF2α Phosphorylation-Dependent Cellular Translation Shutoff *via* PERK/GCN2

**DOI:** 10.3389/fmicb.2021.624540

**Published:** 2021-04-12

**Authors:** Yuanzhi Liu, Anchun Cheng, Mingshu Wang, Sai Mao, Xumin Ou, Qiao Yang, Ying Wu, Qun Gao, Mafeng Liu, Shaqiu Zhang, Juan Huang, Renyong Jia, Dekang Zhu, Shun Chen, Xinxin Zhao, Yanling Yu, Yunya Liu, Ling Zhang, Bin Tian, Leichang Pan

**Affiliations:** ^1^Institute of Preventive Veterinary Medicine, Sichuan Agricultural University, Chengdu, China; ^2^Key Laboratory of Animal Disease and Human Health of Sichuan Province, Sichuan Agricultural University, Chengdu, China; ^3^Research Center of Avian Disease, College of Veterinary Medicine, Sichuan Agricultural University, Chengdu, China

**Keywords:** duck hepatitis A virus type 1, eIF2α, PERK, GCN2, translation shutoff

## Abstract

Duck hepatitis A virus type 1 (DHAV-1) is one of the most deadly pathogens that endanger the duck industry. Most viruses usually turn off host translation after infection to facilitate viral replication and translation. For the first time report to our knowledge, DHAV-1 can induce eIF2α phosphorylation and inhibit cellular translation in duck embryo fibroblasts (DEFs). Moreover, the activity of DHAV-1 in the cells caused obvious eIF2α phosphorylation, which has nothing to do with the viral protein. Subsequently, we screened two kinases (PERK and GCN2) that affect eIF2α phosphorylation through inhibitors and shRNA. Notably, the role of GCN2 in other picornaviruses has not been reported. In addition, when the phosphorylation of eIF2α induced by DHAV-1 is inhibited, the translation efficiency of DEFs restores to a normal level, indicating that DHAV-1 induced cellular translation shutoff is dependent on eIF2α phosphorylation.

## Introduction

Duck viral hepatitis (DVH) is a rapidly spreading and highly lethal infectious disease caused by Duck hepatitis A virus (DHAV) in ducklings, which is very harmful to the duck industry. DHAV includes three genotypes: DHAV-1, DHAV-2, and DHAV-3. Among them, DHAV-1 is more pathogenic. It mainly infects ducklings of 1–4 weeks old and can cause more than 90% mortality. The main feature is liver swelling, bleeding spots, and neurological symptoms (Xie et al., 2018). DHAV-1 belongs to *Avihepatovirus* genus of *Picornaviridae* family. Its genome is about 7,700 nt and consists of a 5' untranslated region (5' UTR), an open reading frame (ORF), a 3' untranslated region (3' UTR), and a poly(A) tail (Liu et al., 2020b). ORF is translated into precursor polyprotein and then cleaved into structural proteins (VP0, VP3, and VP1) and non-structural proteins (2A, 2B, 2C, 3A, 3B, 3C, and 3D). These viral proteins are involved in regulating the life activities of the host and virus (Cao et al., 2016; Sun et al., 2017; Zhang et al., 2017; Yang et al., 2018b; Lai et al., 2019).

After picornavirus infects host cells, it translates viral proteins by hijacking or disrupting cell translation-related factors (PABP, eIF4G, eIF4E, and eIF2; Gingras et al., 1996; Welnowska et al., 2011; Kobayashi et al., 2012; Sun et al., 2017; Yang et al., 2018a). Among them, eIF2 plays an important role in virus infection (Liu et al., 2020a). Under normal circumstances, the GTP conversion factor eIF2B can convert inactive eIF2-GDP into active eIF2-GTP, and active eIF2 mediates the binding of Met-tRNAi^Met^ to the ribosomal 40S subunit in a GTP-dependent manner to initiate peptide chain synthesis. However, eIF2 activity is regulated by phosphorylation of its α subunit S51. Once eIF2α is phosphorylated, eIF2 competitively binds to eIF2B, and the function of eIF2B to convert eIF2-GDP to eIF2-GTP is weakened or disappeared, resulting in GTP that cannot recycle, which ultimately leads to translational inhibition. The four kinases (PERK, GCN2, PKR, and HRI) reported so far phosphorylate eIF2α by sensing different signals, thus regulating the cellular translation process. After picornaviruses invade cells, the accumulation of viral proteins in the endoplasmic reticulum and the production of double-stranded RNA during viral replication activate PERK and PKR, respectively (Jheng et al., 2010; Chang et al., 2017). In addition, GCN2 also plays an antiviral effect in RNA virus infection (Berlanga et al., 2006). HRI is rarely reported in viral infections. It is well-known that PERK, GCN2, and PKR play an important role in viral infection.

The PERK/PKR-eIF2α signaling pathway has been extensively studied in other picornaviruses, but DHAV-1 has not been reported on this aspect. Therefore, the study of DHAV-1 is necessary to reveal the common characteristics of picornaviruses. In this report, we proved that DHAV-1 could cause eIF2α phosphorylation in duck embryo fibroblasts (DEFs) and inhibit cell translation. Moreover, we found that the activity of DHAV-1 in cells rather than viral protein is the cause of obvious eIF2α phosphorylation, and two kinases (PERK and GCN2) are involved in the eIF2α phosphorylation process. Besides, eIF2α phosphorylation inhibition can restore DEFs translation, which indicates that DHAV-1 inhibits DEFs translation through eIF2α phosphorylation.

## Materials and Methods

### Cells and Viruses

The DHAV-1 H strain (GenBank: JQ301467.1) was provided by the Institute of Preventive Veterinary Medicine at Sichuan Agricultural University. The primary DEFs were described previously (Xie et al., 2019; Lai et al., 2020). Six-well cell culture plates were seeded 5 × 10^6^ cells per well and the cells were grown in minimum essential medium (MEM) containing 10% newborn calf serum (Gibco) and incubated at 37°C with 5% CO_2_ in an incubator. Then, DEFs were infected with DHAV-1 for 2 h, and the unbound virus was removed by washing with phosphate-buffered saline (PBS) twice before the cells were overlaid with MEM containing 2% newborn calf serum. DEFs were transfected expression plasmids or poly(I:C) with *TransIntro*™ EL Transfection Reagent (TransGen Biotech). UV-DHAV-1 is obtained by irradiating DHAV-1 with UV light with a wavelength of 253.7 nm for 6 h.

### Antibodies and Reagents

Mouse anti-puromycin antibody was obtained from Millipore. Rabbit anti-phospho-eIF2α (S51) and mouse anti-Myc antibody were obtained from Cell Signaling Technology. Mouse anti-HA and mouse anti-Flag antibody were purchased from MBL. Mouse anti-GFP antibody was obtained from ABclonal. Rabbit anti-beta (β)-actin antibody was obtained from Proteintech. HRP-conjugated goat anti-mouse IgG and HRP-conjugated goat anti-rabbit IgG were purchased from Beyotime. Rabbit anti-VP3 antibody was prepared in our laboratory (Shen et al., 2016).

C16 (PKR Inhibitor) and GSK2606414 (PERK inhibitor) were purchased from APExBIO, and GCN2-IN-1 (GCN2 inhibitor) was purchased from MCE. These inhibitors were dissolved in dimethyl sulfoxide (Sigma) and configured to 10 mmol/L, which were diluted to working concentration with MEM when used. Sodium arsenite was purchased from Sigma. Poly(I:C) was purchased from Invivogen.

### Expression Plasmids

According to the manufacturer’s instructions, to construct plasmids expressing the viral protein, DHAV-1 RNA was isolated using RNAiso Plus Reagent (TaKaRa). According to the manufacturer’s instructions, genomic DNA was then removed, and reverse transcription was performed using a PrimeScriptTM RT Reagent Kit (TaKaRa). VP0, VP1, 2A, 2B, 2C, 3AB, and 3D sequences were amplified from DHAV-1 cDNA with PCR and primers (Table 1). VP0, VP1, 2A, 2B, 2C, and 3D were integrated into the pCAGGs vector with a one-step cloning kit (Vazyme). 3AB was cloned into the pCMV-Myc vector with the DNA Ligation Kit (TaKaRa). The eukaryotic expression vector pCAGGs was gifted by Shanghai Veterinary Research Institute, Chinese Academy of Agricultural Sciences. The eukaryotic expression vector pCMV-Myc was purchased from TaKaRa. pCAGGs-VP3-Flag and pEGFP-N1-3C were also stocks in our laboratory (Lai et al., 2019; Sun et al., 2019). Duck-derived G3BP1 gene was cloned into the pEGFP-C2 vector with the DNA Ligation Kit (TaKaRa). The pEGFP-C2 vector was purchased from YouBio.

**Table 1:** Primers used in this study.

Primers	Forward (5'-3')	Reverse (5'-3')	References
pCAGGs-VP0-Flag	CATCATTTTGGCAAAGAATTCACCGCCACCATGGATACTCTTACCAAAAA	TTGGCAGAGGGAAAAAGATCTTTACTTATCGTCGTCATCCTTGTAATCCTGATTGTCAAATGGTC	New
pCAGGs-VP1-Flag	CATCATTTTGGCAAAGAATTCACCGCCACCATGGGTGATACCAACCAGCT	TTGGCAGAGGGAAAAAGATCTTTACTTATCGTCGTCATCCTTGTAATCTTCAATTTCCAGATCGA	New
pCAGGs-VP3-Flag	CATCATTTTGGCAAAGAATTCGCCACCATGGGAAAGAGAAAACCATGCAGG	TTGGCAGAGGGAAAAAGATCTTCACTTATCGTCGTCATCCTTGTAATCTTGATTGTTAGTTGCCATCTGC	Lai et al., 2019
pCAGGs-2A-HA	CATCATTTTGGCAAAGAATTCACCGCCACCATGGCCTCTGACCAAATTAGAA	TTGGCAGAGGGAAAAAGATCTTTAAGCGTAGTCTGGGACGTCGTATGGGTATTGGTCTGTAGTGATTT	New
pCAGGs-2B-HA	CATCATTTTGGCAAAGAATTCGCCACCATGGCCTCATTTCCAGGTAAAGATGC	TTGGCAGAGGGAAAAAGATCTTCAAGCGTAGTCTGGGACGTCGTATGGGTATTGATCCTCTAACATGTCATTG	New
pCAGGs-2C-HA	CATCATTTTGGCAAAGAATTCACCGCCACCATG TCTGGCAAAACCACCTCTCCT	TTGGCAGAGGGAAAAAGATCTCTAAGCGTAATCTGGAACATCGTATGGGTACTGGTTCATAAAGGAAG	New
pCMV-Myc-3AB	CCGGAATTCCGTCTAAGGTGAGGCGTTTCTCT	CGGGGTACCCTATTCCAATCCAGTTTCTAATT	New
pEGFP-N1-3C	GAATTCTTATGAGCGGGCGGGTGAATTTCAGACATA	GGATCCGGTTGATTAAAAACTGGAAAGACCCTA	Sun et al., 2019
pCAGGs-3D-HA	CATCATTTTGGCAAAGAATTCACCGCCACCATGGGGAAAGTAGTGAGCAA	TTGGCAGAGGGAAAAAGATCTTTAAGCGTAGTCTGGGACGTCGTATGGGTAGATCATCATGCAAGCTG	New
pEGFP-C2-G3BP1	GGAAGATCTCGATGGTGATGGAGAAGCCAAG	CCGGAATTCTCACTGGCGTTGCCCGATCC	New
PKR (Gene ID: 110353866)	GGGAACCGAGGAACA	CGAATGCCGAAAGAAT	New
PERK (Gene ID: 101800258)	CAGCAATGGAGCACTTTCGG	TGGGGATGGAAGAGTTTGCG	New
GCN2 (Gene ID: 101793133)	CAGACCTCGGAAGTTAGA	TACGGAAGTATGGTTCAAAT	New
β-actin (Gene ID: 101800437)	TACGCCAACACGGTGCTG	GATTCATCATACTCCTGCTTG	Soman et al., 2009

### shRNA-Mediated Knockdown of PERK or GCN2

The pGPU6/GFP/Neo-PERK#1/#2/#3 expression vectors and the pGPU6/GFP/Neo-GCN2#1/#2/#3 expression vectors were designed and constructed by GenePharma (Shanghai, China), and the sequences of shRNAs were as follows: PERK#1 5'-GCCAGTCATTAGAGGAAATTT-3', PERK#2 5'-GGCATGATAATGCAATTATTC-3', PERK#3 5'-GCAGGAAAGAGAACCTTAAAG-3', GCN2#1 5'-GCCTAAAGTTAGTGTTATAGC-3', GCN2#2 5'-GGACTATGATGAGTCAAATAT-3', GCN2#3 5'-GGTGCGAAATAAGCTTGATGG-3'. The DEFs were plated into six-well cell culture dishes and cultured overnight. The cells were then transfected with the recombinant plasmids using *TransIntro*™ EL Transfection Reagent (TransGen Biotech).

### RT-PCR Analysis

Total RNA was isolated using RNAiso Plus Reagent (TaKaRa) according to the manufacturer’s instructions. The number of viral copies in total RNA was measured using methods previously established in our laboratory (Hu et al., 2016). Three genes (PKE, PERK, and GCN2) and a housekeeping gene (β-actin) were analyzed by qPCR using primers designed with Primer Premier 5 (Table 1). The expression levels of four genes were determined by qPCR using a SYBR® Premix Ex Taq™ II (Tli RNaseH Plus) Kit (TaKaRa) and an Applied CFX96 Real-Time PCR Detection System (Bio-Rad). Amplification was performed in 10 μl reaction volumes containing 0.5 μl of each primer and 1 μl of cDNA. The following thermal cycling conditions were used: initial activation at 95°C for 30 s, 40 cycles of denaturation at 95°C for 5 s and annealing and extension at 56.9°C for 30 s, and a dissociation curve analysis step.

### Drug and Inhibitor Treatment

Refer to the previous method, DEFs were treated with 1 μg/ml of puromycin for 30 min to detect the protein translation efficiency (Wang et al., 2020). After DHAV-1 infection for 22 h, DEFs were treated with different concentrations of C16, GSK2606414, and GCN2-IN-1 for 2 h to screen the kinases that affect eIF2α phosphorylation. DEFs were treated with different concentrations of sodium arsenite for 30 min to explore the relationship between eIF2α phosphorylation and translation shutoff.

### Western Blot Analysis

Cells were lysed in 200 μl cell lysis buffer (Beyotime) containing 1% PMSF. The cell lysate was centrifuged, and the supernatant was collected. Samples were fractionated by SDS-PAGE electrophoresis and then transferred to PVDF membrane, which was blocked with 5% non-fat dry milk at room temperature for 5–6 h. The membranes were incubated overnight at 4°C with primary antibodies diluted in blocking buffer. The membranes were washed three times with TBS-Tween and incubated for 1 h at 37°C with the respective secondary antibodies diluted in blocking buffer. The membranes were then washed three times with TBS-Tween, and bound proteins were detected using an ECL chromogenic kit (Beyotime).

### Indirect Immunofluorescence

The cells were rinsed (three times) with phosphate-buffered saline (PBS) and then fixed in 4% paraformaldehyde overnight. The cells were permeabilized (0.2% Triton X-100 for 25 min), incubated with blocking solution [5% bovine serum albumin (BSA) in PBS with Tween 20 (PBST) for 60 min at 37°C], and then rinsed with PBST. The cells were then treated with DAPI (Roche). Images were captured using an 80i upright microscope (Nikon) and a SPOT Flex camera.

### Cell Viability Assays

The cell viability was measured using the Cell Counting Kit-8 (Biosharp) according to the manufacturer’s instructions.

## Results

### DHAV-1 Can Induce eIF2α Phosphorylation and Inhibit Cellular Translation in DEFs

After many viruses infect host cells, they hijack the cell’s translation-related factors and reduce the overall cellular translation efficiency. Puromycin is a protein synthesis inhibitor. It has a structure similar to the end of the tRNA molecule. It can replace the aminoacylated tRNA to enter the ribosome A site and bind to the elongating polypeptide chain. When the elongating polypeptide is transferred to this abnormal A position, the polypeptide synthesis is blocked, and the immature C-terminal polypeptide containing puromycin is released. Therefore, we use the puromycin antibody to detect labeled elongating polypeptides, which can dynamically detect protein translation efficiency in a real-time manner. And, this method has been used in our previous report (Wang et al., 2020).

**Figure 1:**
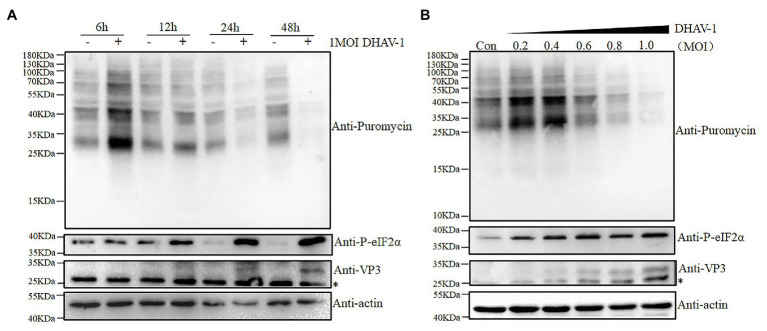
The effect of virus infection on the eIF2α phosphorylation and translation in duck embryo fibroblasts (DEFs). **(A)** DEFs were infected with Duck hepatitis A virus type 1 (DHAV-1) at MOI of 1. The cells were treated with puromycin (1 μg/ml; 30 min) at 6, 12, 24, and 48 h after infection and harvested for immunoblot analysis. **(B)** DEFs were infected with DHAV-1 at MOI of 0.2, 0.4, 0.6, 0.8, 1.0, respectively. The cells were treated with puromycin (1 μg/ml; 30 min) at 24 h after infection and harvested for immunoblot analysis. The bands marked by asterisk (*) are non-specific proteins.

DEFs were infected with DHAV-1 at MOI = 1, and samples were harvested at 6, 12, 24, and 48 h after infection, and control groups were set at each time point. Before harvesting the cells, puromycin (1 μg/ml) was added to the medium, and the cells were incubated at 37°C for 30 min (Wang et al., 2020). As shown in Figure 1A, after DHAV-1 infects DEFs, eIF2α phosphorylation increases with the infection time, while DEFs translation is gradually inhibited. Subsequently, to further prove the effectiveness of DHAV-1 on eIF2α phosphorylation and cellular translation, DEFs were infected with DHAV-1 at 0.2 MOI, 0.4 MOI, 0.6 MOI, 0.8 MOI, and 1.0 MOI, and samples were collected at 24 hpi, and the control group was set at the same time. As shown in Figure 1B, eIF2α phosphorylation increases with the increase of the infectious dose of DHAV-1, while the overall translation of DEFs is gradually inhibited. Interestingly, the viral protein VP3 can still be expressed (Figures 1A,B), indicating that the inhibition of DEFs translation does not inhibit the translation of DHAV-1. This is related to the ability of the internal ribosome entry site (IRES) element of DHAV-1 to initiate cap-independent translation (Liu et al., 2011), and this result is similar to poliovirus (PV; Kastan et al., 2020). The above results show that DHAV-1 infection can induce eIF2α phosphorylation and inhibit cellular translation in DEFs.

### DHAV-1 Induces SGs Formation in Infected Cells

**Figure 2:**
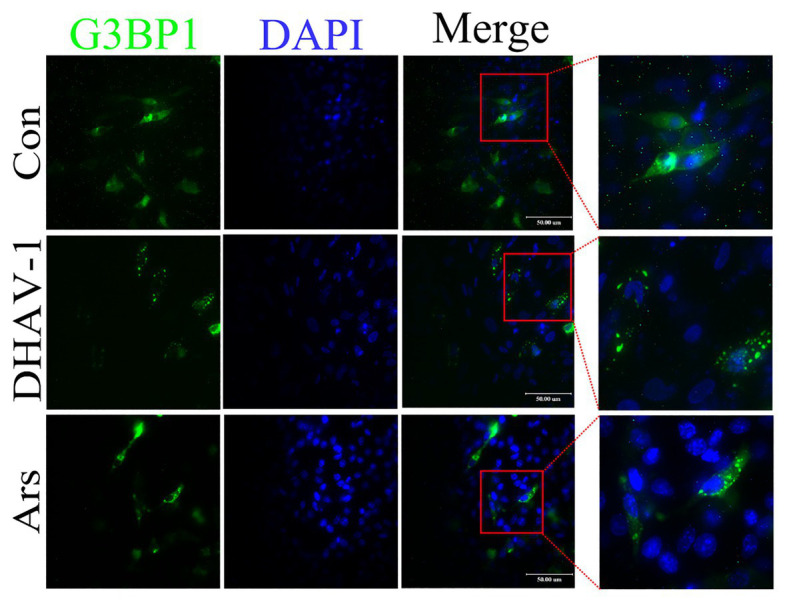
DHAV-1 induces SGs formation in infected cells. DEFs were transfected with recombinant plasmid pEGFP-C2-G3BP1. Then, at 12 h after transfection, DEFs were inoculated with DHAV-1 at MOI = 1 for 24 h. In the Ars group, DEFs were treated with 700 μM sodium arsenite for 30 min. The expression and distribution of EGFP-G3BP1 were observed with a fluorescence microscope. Scale bar, 50 μm.

Phosphorylation of eIF2α causes total translation stagnation in the cells. The stalled messenger ribonucleoprotein (mRNP) complex aggregates under viral infection stress to form stress granules (SGs). Ras GTPase-activating protein-binding protein 1 (G3BP1) can regulate the SG core network through positive or negative cooperativity with other G3BP1-binding factors (Yang et al., 2020). Therefore, G3BP1 is often used as a marker protein for SGs. Due to lack of duck-derived antibody against G3BP1, we cloned duck-derived G3BP1 gene into pEGFP-C2 vector. As shown in Figure 2, in the control group, EGFP-G3BP1 was uniformly distributed in the cytoplasm. However, EGFP-G3BP1 displayed an obvious granular pattern in the cytoplasm of DHAV-1-infected cells, which was similar to the Ars group. These results indicate that DHAV-1 induces SGs formation in infected cells.

### The Viral Protein of DHAV-1 Is Not the Cause of Significant eIF2α Phosphorylation

**Figure 3:**
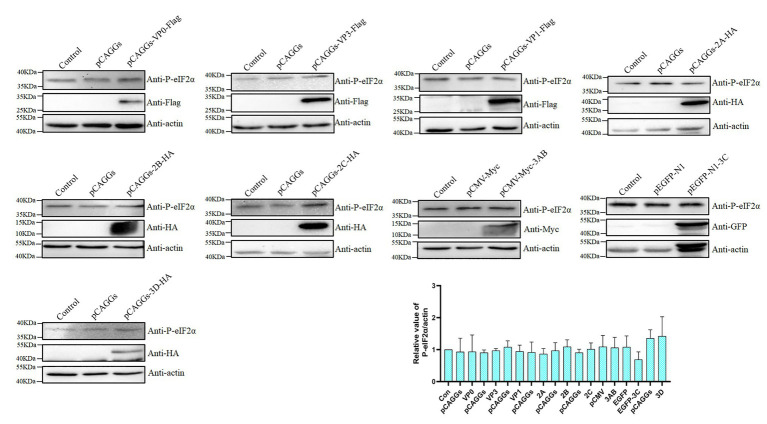
The viral protein is not the cause of significant eIF2α phosphorylation. DEFs were transfected with plasmids expressing the viral protein. The cells were harvested at 36 h after transfection and immunoblot analysis with the indicated antibodies.

It was previously reported that the foot-and-mouth disease virus VP2 could cause the phosphorylation of eIF2α (Sun et al., 2018), so we speculate whether a certain viral protein of DHAV-1 is the cause of phosphorylation of eIF2α. DEFs were transfected with recombinant plasmids of structural proteins (pCAGGs-VP0-Flag, pCAGGs-VP3-Flag, and pCAGGs-VP1-Flag) and non-structural proteins (pCAGGs-2A-HA, pCAGGs-2B-HA, pCAGGs-2C-HA, pCMV-Myc-3AB, pEGFP-N1-3C, and pCAGGs-3D-HA) of DHAV-1. As shown in Figure 3, compared to the empty vector group, the expression of VP0, VP1, 2A, 3AB, 3C, and 3D proteins did not increase eIF2α phosphorylation. The expression of VP3, 2B, and 2C proteins can slightly increase eIF2α phosphorylation, which may be related to the function of VP3, 2B, and 2C proteins in cells (Cong et al., 2016; Li et al., 2016; Lai et al., 2019). However, none of these viral proteins caused eIF2α phosphorylation as obvious as DHAV-1 infection. These results indicate that viral protein is not the cause of significant eIF2α phosphorylation.

### UV-Inactivated DHAV-1 No Longer Phosphorylates eIF2α

**Figure 4:**
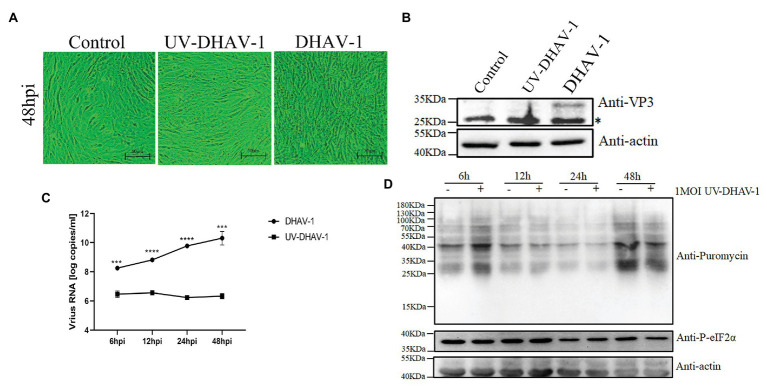
Viral activity in DEFs is the cause of eIF2α phosphorylation. **(A)** CPEs in DEFs at 48 hpi. Scale bar, 50 μm. **(B)** The expression of VP3 protein in DEFs at 48 hpi. The bands marked by asterisk (*) are non-specific proteins. **(C)** DEFs were infected with DHAV-1 and UV-DHAV-1 at MOI of 1, respectively. The X-axis shows the different time points, and the Y-axis represents the logarithm of the number of viral RNA copies. **(D)** DEFs were infected with UV-DHAV-1 at MOI of 1. The cells were treated with puromycin (1 μg/ml; 30 min) at 6, 12, 24, and 48 h after infection and harvested for immunoblot analysis. Differences between the two groups were analyzed using Student’s *t*-test and considered as significant at ^***^p < 0.001 and ^****^p < 0.0001.

The previous results have shown that the viral protein of DHAV-1 is not the cause of obvious phosphorylation of eIF2α. To further investigate whether the viral activity is the cause of eIF2α phosphorylation, we used UV to inactivate DHAV-1 and then infected DEFs at 1 MOI. As shown in Figures 4A–C, compared to the control group, the UV-inactivated DHAV-1 did not appear obvious CPEs (cells shrink, round, and fragment), no VP3 expression was detected, and no increase in virus copy number, indicating that the UV-inactivated DHAV-1 lost the ability to replicate. Compared with the control group, UV-inactivated DHAV-1 did not cause the phosphorylation of eIF2α and did not affect the translation of DEFs (Figure 4D). These results indicate that UV-inactivated DHAV-1 no longer phosphorylates eIF2α, which is similar to EV71 (Jheng et al., 2010).

### PERK and GCN2 Are Involved in eIF2α Phosphorylation During DHAV-1 Infection

**Figure 5:**
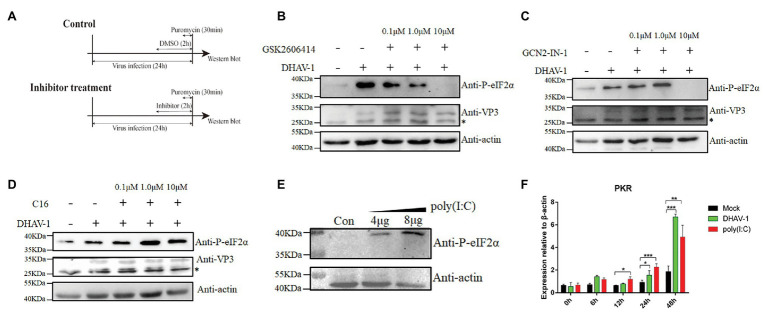
PERK and GCN2 are involved in eIF2α phosphorylation during DHAV-1 infection. **(A)** Screen the kinases that affect eIF2α phosphorylation. DEFs were infected with DHAV-1 at MOI of 1. After 22 h of infection, different concentrations of kinase inhibitors were added to DEFs for 2 h. Then, DEFs were harvested for immunoblot analysis with the indicated antibodies. **(B)** PERK inhibitor GSK2606414 inhibits eIF2α phosphorylation induced by DHAV-1. **(C)** GCN2 inhibitor GCN2-IN-1 inhibits eIF2α phosphorylation induced by DHAV-1. **(D)** PKR inhibitor C16 cannot inhibit eIF2α phosphorylation induced by DHAV-1. **(E)** Transfection of poly(I:C) activate PKR kinase. **(F)** DHAV-1 and poly(I:C) stimulate PKR transcription. Differences between two groups were analyzed using Student’s *t*-test and considered as significant at ^*^p < 0.05, ^**^p < 0.01, and ^***^p < 0.001. The bands marked by asterisk (*) are non-specific proteins.

Four known kinases phosphorylate eIF2α in cells, namely, PERK, PKR, GCN2, and HRI. However, we only found three kinases PERK, PKR, and GCN2, in duck-derived cells from National Center for Biotechnology Information (NCBI). To better understand which kinase plays a role during DHAV-1 infection, we used PERK, PKR, and GCN2 kinase inhibitors for screening. Due to the lack of antibodies against PERK, PKR, and GCN2, we can only indirectly reflect the three kinases’ activation through the phosphorylation of eIF2α. DEFs were infected with DHAV-1 at 1 MOI. At 22 h after infection, PERK, PKR, and GCN2 kinase inhibitors were added, and cells were harvested after 2 h of treatment (Figure 5A). After adding PERK inhibitor (GSK2606414) and GCN2 inhibitor (GCN2-IN-1), eIF2α phosphorylation was inhibited, indicating that PERK and GCN2 are involved in DHAV-1 induced eIF2α phosphorylation (Figures 5B,C). However, PKR inhibitor (C16) did not inhibit eIF2α phosphorylation, which indicates that PKR is not involved in eIF2α phosphorylation induced by DHAV-1 (Figure 5D). To show whether the PKR kinase of DEFs can be activated, DEFs were transfected with poly(I:C), and then eIF2α phosphorylation and PKR transcription levels were detected. As shown in Figures 5E,F, poly(I:C) can cause the phosphorylation of eIF2α and increase PKR transcription.

**Figure 6:**
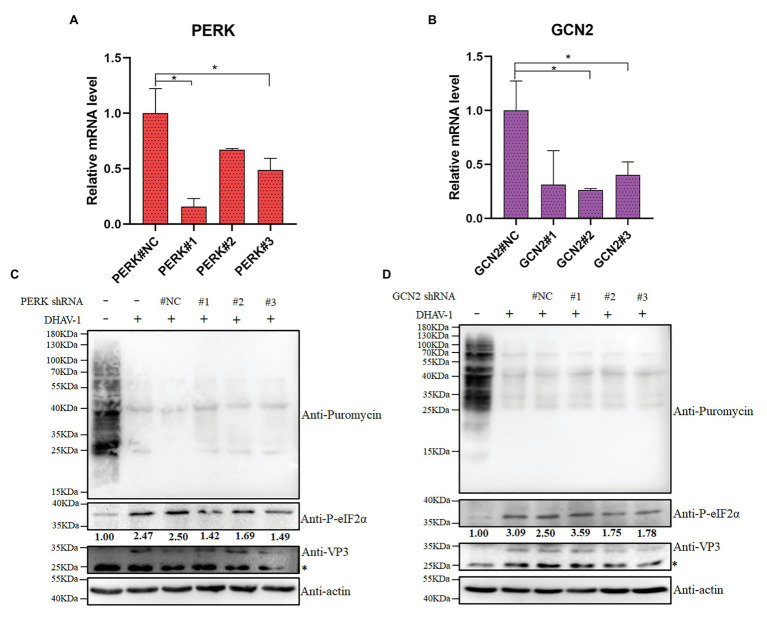
The effect of PERK or GCN2 knock-down on eIF2α phosphorylation and cell translation. **(A,B)** The knock-down efficiency of shRNA is measured by quantitative RCR. DEFs were transfected with shRNA. Then, at 36 h after transfection, DEFs were harvested for qPCR according to the manufacturer’s instructions. Differences between two groups were analyzed using Student’s *t*-test and considered as significant at ^*^p < 0.05. **(C,D)** DEFs were transfected with pGPU6/GFP/Neo-PERK#NC/#1/#2/#3 expression vectors or pGPU6/GFP/Neo-GCN2#NC/#1/#2/#3. Then, at 12 h after transfection, DEFs were inoculated with DHAV-1 at MOI = 1 for 24 h. DEFs were treated with puromycin (1 μg/ml; 30 min) and harvested for immunoblot analysis. The bands marked by asterisk (*) are non-specific proteins.

In order to exclude the non-specific effects of inhibitors, we used shRNA to knock down PERK or GCN2. We designed three shRNAs against PERK or GCN2 and found that shRNAs could interfere with the transcription of PERK or GCN2 (Figures 6A,B). We also found that DHAV-1-induced the phosphorylation of eIF2α was reduced after PERK or GCN2 knockdown (Figures 6C,D). These results indicate that PERK and GCN2 are involved in eIF2α phosphorylation during DHAV-1 infection.

### DHAV-1 Inhibits Cell Translation Through eIF2α Phosphorylation

eIF2 plays an important role in eukaryotic cells, and its alpha subunit phosphorylation controls the cellular translation initiation. Many reports indicate that the virus causes cell translation inhibition through eIF2α phosphorylation, but recently dengue virus (DENV) and mouse norovirus (MNV) have found that cell translation inhibition and eIF2α phosphorylation are not coupled with each other (Roth et al., 2017; Brocard et al., 2020). Since the transfection efficiency of DEFs is not high, the knockdown effect of shRNA is not very good. In the Figures 6C,D, after we knocked down PERK or GCN2 using shRNA, eIF2α phosphorylation was weakened, but the cellular translation level did not increase. The main reason is that the level of the GTP conversion factor eIF2B in the cell is 10–20 times lower than that of eIF2; therefore, small changes in eIF2 phosphorylation can have a significant effect on protein translation (Montero et al., 2008).

**Figure 7:**
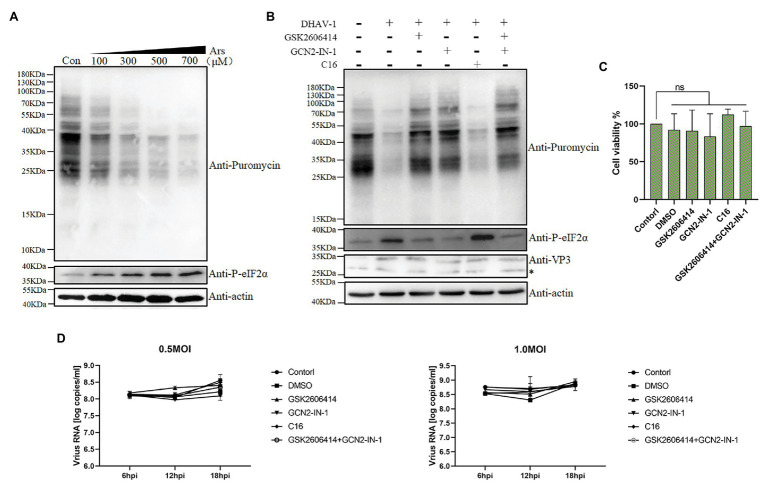
DHAV-1 inhibits cellular translation through eIF2α phosphorylation. **(A)** DEFs were treated with different concentrations of sodium arsenite for 30 min. Then, DEFs were treated with puromycin (1 μg/ml; 30 min) and harvested for immunoblot analysis. **(B)** DEFs were infected with DHAV-1 at MOI of 1. After 22 h of infection, DEFs were treated with kinase inhibitors for 2 h, respectively. Then, the cells were treated with 1 μg/ml puromycin for 30 min. The bands marked by asterisk (*) are non-specific proteins. **(C)** Cell viability of DEFs treated with inhibitors. **(D)** The effect of kinase inhibitors on DHAV-1 propagation.

To explore the direct relationship between eIF2α phosphorylation and DEFs translation inhibition, we used sodium arsenite (a reagent known to induce phosphorylation of eIF2α) as a positive control. As shown in the Figure 7A, sodium arsenite can induce phosphorylation of eIF2α very well. Using puromycin to label cells, we found that the translation efficiency of cells decreased. This phenomenon is consistent with the result in Figure 1, which indicates that DHAV-1 may inhibit DEFs translation through phosphorylation of eIF2α.

The results described above did not provide a clear answer to the role of eIF2α phosphorylation in translation shutoff. Therefore, we used inhibitors to treat DEFs and then labeled them with puromycin for 30 min to test whether the translation inhibition of DEFs was restored (Figure 5A). As shown in Figures 7B,C after treating DEFs with 10 μM PERK or 10 μM GCN2 inhibitors, the phosphorylation of eIF2α caused by DHAV-1 was reduced, consistent with the previous results (Figures 5B,C). And after the inhibitor treatment, the translation of the cells was restored, which was close to the mock group level. Besides, to ensure that the same inhibitor dose is added, we use 5 μM PERK and 5 μM GCN2 inhibitors to treat DEFs simultaneously. The results showed that the phosphorylation of eIF2α also recovered to the mock group level, and translation inhibition was restored. However, treatment of DEFs with PKR inhibitor did not restore eIF2α phosphorylation as in Figure 5D, nor did it restore the translation level. These results above show that DEFs translation shutoff caused by DHAV-1 is caused by phosphorylation of eIF2α.

Subsequently, we determined the effect of these kinase inhibitor treatments on DHAV-1 propagation. We treated DEFs with corresponding concentrations of kinase inhibitors for 2 h and then infected DEFs with 0.5 MOI or 1.0 MOI DHAV-1, respectively. The virus copy number was measured at 6, 12, and 18 h after infection, and the results showed that treatment with these kinase inhibitors would not affect DHAV-1 propagation (Figure 7D).

## Discussion

mRNA undergoes translation initiation, elongation, and post-translational modification to form a protein with a specific structure and function. Since eIF2 plays an important regulatory role in the translation initiation, inhibition of host cell translation by eIF2α phosphorylation is a common strategy for most viruses, but recently DENV and MNV have also reported translation shutoff independent of eIF2α phosphorylation. Therefore, to explore whether the translation shutoff of DEFs caused by DHAV-1 is dependent on eIF2α phosphorylation. After using inhibitors to weaken eIF2α phosphorylation, we found that DEFs infected with DHAV-1 recovered to the same translation level as the mock group, indicating that DHAV-1 inhibits DEFs translation by phosphorylation of eIF2α (Figures 1, 7B). In addition, we found that DHAV-1 induces SGs formation (Figure 2).

Under stress, eIF2α phosphorylation leads to an increase in the affinity of eIF2 and eIF2B, eIF2 competitively binds to eIF2B, and the function of eIF2B to convert eIF2-GDP to eIF2-GTP is weakened or disappeared. Therefore, how the virus synthesizes its protein in the presence of eIF2α phosphorylation is an interesting question. The accessory protein AcP10 of beluga whale coronavirus (Bw-CoV) can bind to eIF2B, hindering the eIF2 binding to eIF2B after eIF2α phosphorylation, thereby promoting the function of eIF2B-mediated conversion of eIF2-GDP to eIF2-GTP (Rabouw et al., 2020). Similarly, the L protein encoded by Aichivirus also has a similar effect to AcP10 (Rabouw et al., 2020). However, as a picornavirus, the DHAV-1 genome does not encode the L protein but has a 3C protein with similar functions to the L protein. And, we found that the 3C protein of DHAV-1 can weakly down-regulate the phosphorylation of eIF2α (Figure 3). We speculate that the 3C protein may have a similar mechanism to the L protein of Aichivirus, but this requires further experimental exploration.

eIF2α phosphorylation plays an important role in viral infection (Liu et al., 2020a). Viruses shutoff cellular translation by eIF2α phosphorylation, reducing the competition between host proteins and viral proteins for ribosomes. Moreover, eIF2α phosphorylation facilitates the selective expression of some host proteins, such as ATF4 and CHOP, which promote autophagy or apoptosis in cells and promote virus proliferation (Harding et al., 2000; Tallóczy et al., 2002; B’Chir et al., 2013; Sun et al., 2018; Isobe et al., 2019). In addition, eIF2α phosphorylation can regulate the cell cycle, providing a favorable environment for virus replication (Wang et al., 2018a,b). In this study, we did not screen any viral protein that significantly increased the phosphorylation of eIF2α (Figure 3) and found that UV-inactivated DHAV-1 no longer phosphorylated eIF2α (Figure 4D). The latter results show that DHAV-1 activates two kinases, PERK and GCN2 (Figures 5, 6). These results indicate that the activity of DHAV-1 in cells is the cause of eIF2α phosphorylation.

The picornavirus infection causes cellular endoplasmic reticulum stress and activates PERK (Jheng et al., 2010; Ranjitha et al., 2020). Our results also show that PERK is involved in eIF2α phosphorylation caused by DHAV-1, indicating that DHAV-1 activates PERK like other picornaviruses (Figures 5B, 6C). Another kind of eIF2α kinase, PKR, has an antiviral effect related to the natural immune pathway (Pham et al., 2016; Yoshida et al., 2017). However, picornaviruses have evolved a series of antagonistic measures to evade the antiviral effects of PKR. For instance, enterovirus 71 (EV71) hydrolyzes PKR through 3C protease (Chang et al., 2017), foot-and-mouth disease virus (FMDV) degrades PKR through the lysosomal pathway (Li et al., 2017), and encephalomyocarditis virus (EMCV) causes PKR dephosphorylation (Ng et al., 2013). In short, these antagonistic measures avoid the activation of PKR. Our experiments show that PKR does not participate in the phosphorylation of eIF2α caused by DHAV-1 (Figures 5D,E). We speculate that this is related to PKR activation inhibition by DHAV-1, which requires further experimental proof. Interestingly, we also demonstrated that GCN2 is involved in DHAV-1 induced eIF2α phosphorylation (Figures 5C, 6D). As mentioned above, the PERK or PKR kinase has been widely reported in other picornaviruses, but the role of GCN2 in picornaviruses has not been reported, which may be due to the lack of screening of these three kinases during the research process. Among other RNA viruses, GCN2 plays an antiviral effect in Sindbis virus (SINV) infection, and GCN2 participates in eIF2α phosphorylation caused by MNV infection (Berlanga et al., 2006; Brocard et al., 2020). Therefore, we hypothesized that DHAV-1 infection with DEFs also causes metabolic stress, which activates GCN2 and phosphorylates eIF2α.

It is a common phenomenon that PERK and GCN2 kinases simultaneously regulate the same substrate (Hamanaka et al., 2005; Krishnamoorthy et al., 2008; You et al., 2018; Jin et al., 2019). We used PERK or GCN2 inhibitor to treat DEFs and found that both inhibitors can attenuate eIF2α phosphorylation, related to the redundancy or even compensation of the two kinases’ functions (Donnelly et al., 2013; You et al., 2018). However, when PERK and GCN2 inhibitors treated DEFs simultaneously, they did not completely abolish eIF2α phosphorylation and only recovered to the same level as the mock group (Figure 7B). This phenomenon may be related to cells requiring eIF2α phosphorylation to maintain cell self-renewal capacity and the synthesis of certain proteins (Zismanov et al., 2016).

In summary, our results show that DHAV-1 induces eIF2α phosphorylation-dependent cellular translation shutoff and that two kinases, PERK, and GCN2, are involved in eIF2α phosphorylation. These results provide basic data for further research on the pathogenic mechanism of DHAV-1.

## Data Availability Statement

The raw data supporting the conclusions of this article will be made available by the authors, without undue reservation.

## Author Contributions

YaL conceived and carried out the experiments, analyzed the data, and wrote the manuscript. AC and MW conceived and supervised the study. SM, XO, QY, YW, QG, ML, SZ, JH, RJ, DZ, SC, XZ, YY, YnL, LZ, BT, and LP interpreted the data and revised the manuscript. All authors contributed to the article and approved the submitted version.

### Conflict of Interest

The authors declare that the research was conducted in the absence of any commercial or financial relationships that could be construed as a potential conflict of interest.
