# Supplementary material for: Duck Hepatitis A Virus Type 1 Induces eIF2α Phosphorylation-Dependent Cellular Translation Shutoff via PERK/GCN2
Source: Front Microbiol. 2021 Apr 12;12:624540. doi: 10.3389/fmicb.2021.624540 (PMC8072014; doi:10.3389/fmicb.2021.624540)
Supplement: Supplementary file 1 [file Data_Sheet_1.PDF]

Supplementary Material

DHAV-1 induces eIF2α phosphorylation-dependent cellular translation shutoff via PERK/GCN2

Yuanzhi Liu[1,2,3¶], Anchun Cheng[1,2,3¶], Mingshu Wang[1,2,3*], Sai Mao[1,2,3], Xumin Ou[1,2,3], Qiao Yang[1,2,3], Ying Wu[1,2,3], Qun Gao[1,2,3], Mafeng Liu[1,2,3], Shaqiu Zhang[1,2,3], Juan Huang[1,2,3], Renyong Jia[1,2,3], Dekang Zhu[2,3], Shun Chen[1,2,3], Xin-Xin Zhao[1,2,3], Yanling Yu[1,2,3], Yunya Liu[1,2,3], Ling Zhang[1,2,3] , Bin Tian[1,3], Leichang Pan[1,3]

Corresponding authors: Mingshu Wang, mshwang@163.com

Supplementary Figures

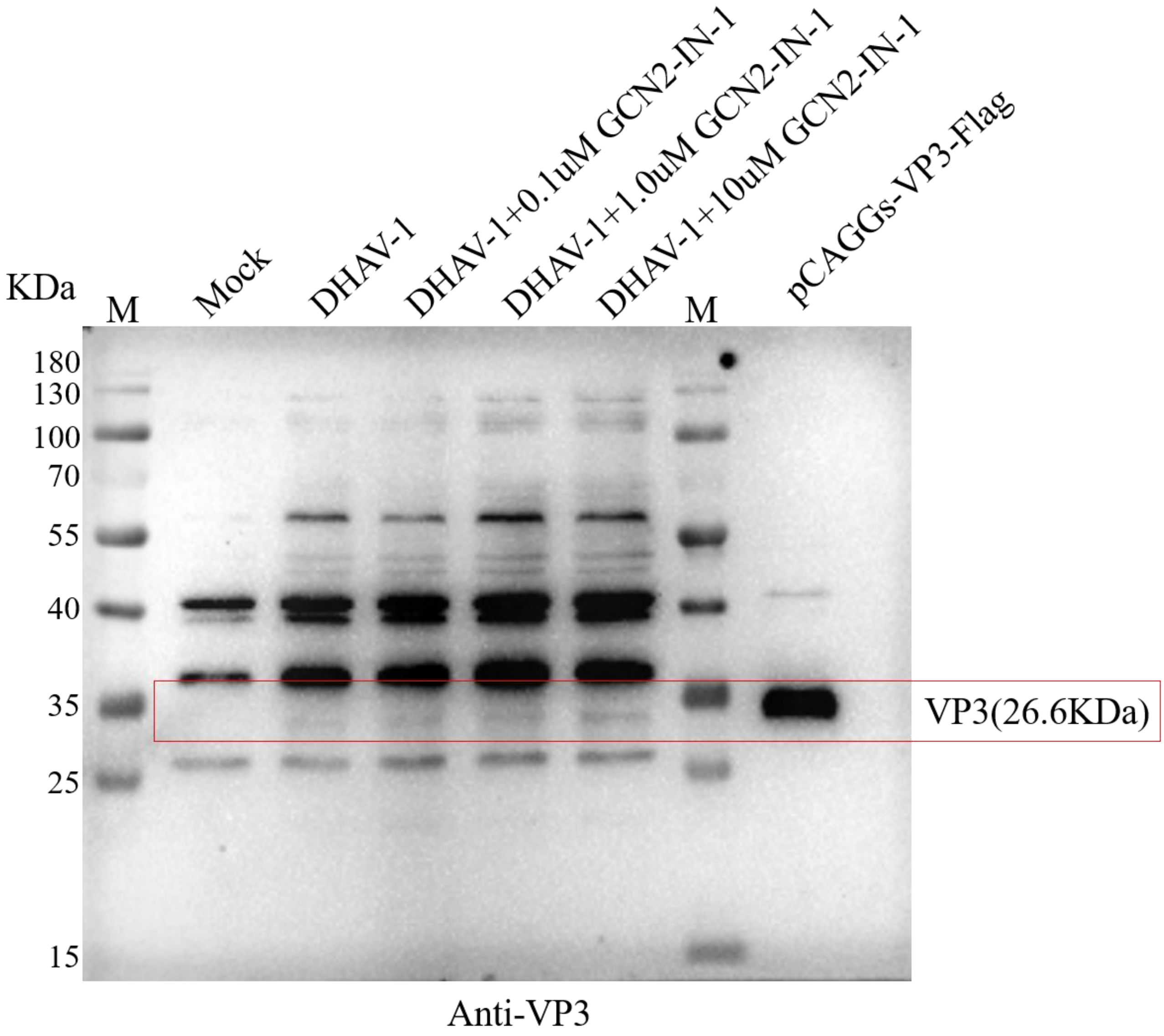

Supplementary Figure 1. The VP3 eukaryotic expression plasmid (pCAGGs-VP3-Flag) confirms the correct region of VP3. DEFs were infected with DHAV-1 at MOI of 1. After 22 h of infection, different concentrations of GCN2 inhibitors (GCN2-IN-1) were added to DEFs for 2 h. Then, DEFs were harvested for immunoblot analysis with rabbit anti-VP3 serum. In order to determine the correct band of VP3, cell sample expressing VP3 protein was used as a positive control. The size of VP3 protein is 26.6KDa, as shown by the red border.
